# Effects of percutaneous transluminal angioplasty and associated factors in access hand oxygenation in patients undergoing hemodialysis

**DOI:** 10.1038/s41598-023-29879-0

**Published:** 2023-02-13

**Authors:** Tomoko Sugiyama, Kiyonori Ito, Susumu Ookawara, Hirofumi Shimoyama, Mitsutoshi Shindo, Momoko Hirata, Hiromi Shimoyama, Yuichi Nakazato, Yoshiyuki Morishita

**Affiliations:** 1 Division of Nephrology, First Department of Integrated Medicine, Saitama Medical Center, Jichi Medical University, 1-847 Amanuma-cho, Omiya-ku, Saitama, Saitama 330-8503 Japan; 2 Division of Nephrology, Yuai Nisshin Clinic, Hakuyukai Medical Corporation, Saitama, Japan; 3 Teikyo University Graduate School of Public Health, Tokyo, Japan; 4 Division of Nephrology, Yuai Clinic, Hakuyukai Medical Corporation, Saitama, Japan

**Keywords:** Kidney diseases, Medical research, Renal replacement therapy

## Abstract

In hemodialysis (HD) patients with arteriovenous fistula (AVF), changes in systemic or peripheral tissue circulation occur non-physiologically via the presence of AVF; however, associations between blood flow and tissue oxygenation in the brain and access hand are uncertain. In this study, 85 HD patients with AVF were included and evaluated for changes in flow volume (FV) and regional oxygen saturation (rSO_2_) in the brain and hands with AVF before and after percutaneous transluminal angioplasty (PTA). Furthermore, we evaluated the factors that determine access hand rSO_2_ without stenosis after PTA. Brachial arterial FV increased after PTA (p < 0.001), and carotid FV decreased (p = 0.008). Access hand rSO_2_ significantly decreased after PTA (p < 0.001), but cerebral rSO_2_ did not significantly change (p = 0.317). In multivariable linear regression analysis of factors associated with access hand rSO_2_, serum creatinine (standardized coefficient: 0.296) and hemoglobin (standardized coefficient: 0.249) were extracted as independent factors for access hand rSO_2_. In conclusion, a decrease in access hand oxygenation and maintenance of cerebral oxygenation were observed throughout PTA. To maintain access hand oxygenation, it is important to adequately manage Hb level and maintain muscle mass, in addition to having an AVF with appropriate blood flow.

## Introduction

An arteriovenous fistula (AVF) is one of the main options for vascular access in hemodialysis (HD) therapy. An AVF is usually created by anastomosing the arterial and venous vessels in the upper extremity, potentially reducing the risk of thrombosis and infection, compared to arteriovenous grafts or long-term indwelling catheters. Therefore, AVF creation is recommended as the first choice of HD access in Japan^1^. However, AVF may present some problems. AVF veins may become stenosed and occluded frequently due to the burden of the pressure from the artery or the effects of vascular puncture for HD therapy. In general, Doppler ultrasonography could be used as a device in the surveillance of AVF for assessing vascular stenosis or function including AVF blood flow or morphology^1^. When an AVF has stenosis, percutaneous transluminal angioplasty (PTA) might be performed, and appropriate PTA allows HD patients to maintain AVF patency and adequate blood flow during HD^1^. Mature AVF, with a blood flow of approximately 500 mL/min and non-physiological circulation^1,2^, may also affect systemic hemodynamics, including cardiac output^3^ or peripheral tissue circulation^4^. In particular, steal syndrome, which can lead to severe ischemia, should be paid attention to in clinical setting of HD facilities.

Recently, near-infrared spectroscopy (NIRS) has been used to measure regional oxygen saturation (rSO_2_). This rSO_2_ measurement could enable non-invasive and real-time monitoring of tissue oxygenation, and has been utilized in various fields, including perioperative patients and newborns^5–7^. In HD patients, cerebral rSO_2_ was reported to be lower than that in healthy controls or patients with chronic kidney disease without dialysis therapy^8,9^, and access hand rSO_2_ was lower than that in healthy controls^10^. However, tissue oxygen dynamics in HD patients still need to be clarified and especially, how the presence of an AVF can affect tissue oxygenation via non-physiological circulation requires further study. Angiostomy between the artery and vein or PTA for the AVF might have a strong impact on blood flow or systemic circulation. However, thus far, reports about changes in tissue oxygenation, including in the brain and access hand, are limited. Therefore, in this study, we aimed to investigate the changes in rSO_2_ values in the brain and access hand before and after PTA, which caused changes in vascular resistance and blood flow at the AVF. Furthermore, we evaluated the factors that determined access hand rSO_2_ without stenosis after PTA.

## Results

**Figure 1:**
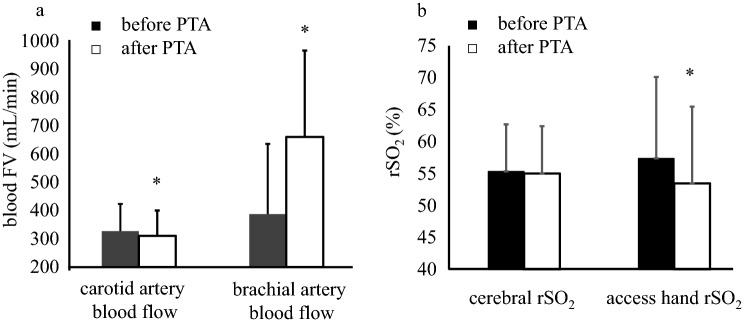
Blood flow and rSO_2_ before and after PTA. (**a**) Comparison of carotid and brachial artery blood flow before and after PTA. (**b**) Comparison of rSO_2_ before and after PTA. Asterisk shows statistically significant compared with before PTA. *PTA* percutaneous transluminal angioplasty, *FV* flow volume, *rSO*_*2*_ regional oxygen saturation.

The differences in clinical parameters measured before and after PTA are summarized in Table 1. Brachial arterial blood flow volume (FV) significantly increased after PTA compared with that before PTA (p < 0.001, Fig. 1a). At the same time, the brachial arterial resistance index (p < 0.001), the carotid blood FV (p = 0.008), and the access hand rSO_2_ (p < 0.001, Fig. 1b) significantly decreased after PTA compared with those before PTA. In contrast, the cerebral rSO_2_ was not significantly different before and after PTA.

**Table 1:** Comparison of clinical parameters between before and after PTA.

Variables	Before PTA	After PTA	p
Systolic blood pressure (mmHg)*	153 (135–174)	150 (132–169)	0.468
Diastolic blood pressure (mmHg)*	75 (65–86)	75 (67–87)	0.739
Heart rate (beats/min)*	69 (60–78)	68 (60–76)	0.021
Brain			
Cerebral rSO_2_ (%)	55.3 ± 7.4	55.0 ± 7.4	0.317
FV at common carotid artery (mL/min)	326.9 ± 96.5	310.3 ± 89.8	0.008
Access hand			
Access hand rSO_2_ (%)	57.4 ± 12.7	53.5 ± 12.0	< 0.001
FV at brachial artery (mL/min)*	343 (236–472)	611(478–773)	< 0.001
RI at brachial artery	0.66 ± 0.13	0.54 ± 0.09	< 0.001
Access hand SpO_2_ (%)*	98 (98–99)	98 (97–99)	0.034

Continuous data are presented as mean ± standard deviation.

*rSO*_*2*_ regional oxygen saturation, *SpO*_*2*_ saturation of percutaneous oxygen, *PTA* percutaneous transluminal angioplasty, *FV* flow volume, *RI* resistance index.

*Wilcoxon signed-rank test for statistical analysis.

Table 2 shows the correlations between patients’ characteristics and access hand rSO_2_ after PTA. Access hand rSO_2_ showed significant and positive correlations with body mass index (BMI), hemoglobin (Hb) level, serum albumin concentration, blood urea nitrogen, and serum creatinine, as well as negative correlations with age and the use of antiplatelet agents. In the multivariable linear regression analysis, serum creatinine (standardized coefficient: 0.296) and Hb levels (standardized coefficient: 0.249) were extracted as independently associated factors for access hand rSO_2_ after PTA (Table 3).

**Table 2:** Correlation between clinical parameters and access hand rSO_2_.

Variables	Access hand rSO_2_	
	r	p
Male/female	0.117	0.285
Age (year)	−0.318^#^	0.001
HD vintage (month)	−0.059^#^	0.589
Primary disease		
Diabetes mellitus	−0.043	0.696
Nephrosclerosis	0.059	0.590
Chronic glomerulonephritis	−0.092	0.403
Others		
Arteriovenous fistula		
Right arm/left arm	0.061	0.580
Medical history		
Diabetes mellitus	−0.012	0.915
Hypertension	0.032	0.773
Dyslipidemia	−0.069	0.529
Coronary artery disease	−0.052	0.639
Cerebrovascular disease	0.104	0.341
Peripheral artery disease	−0.112	0.307
Medication		
Antiplatelet agents	−0.256	0.018
Anticoagulant	−0.132	0.227
Antihypertensive drug	0.014	0.902
Vital signs and physiological findings after PTA		
SBP (mmHg)	0.153^#^	0.163
DBP (mmHg)	−0.010^#^	0.930
Heart rate (beats/min)	−0.052^#^	0.640
SpO_2_ (%)	0.075^#^	0.495
BMI (kg/m^2^)	0.252	0.022
Echography findings after PTA		
FV at brachial artery (mL/min.)	0.165^#^	0.131
RI at brachial artery	0.009	0.938
Laboratory findings		
Hemoglobin (g/dL)	0.432	< 0.001
Albumin (g/dL)	0.372	< 0.001
Blood urea nitrogen (mg/dL)	0.216^#^	0.047
Creatinine (mg/dL)	0.408	< 0.001
Na (mEq/L)	0.079	0.474
K (mEq/L)	−0.020	0.859
Ca (mg/dL)	0.097	0.379
P (mg/dL)	0.027^#^	0.804

Categorical data are presented as number (%), continuous data are presented as mean ± standard deviation.

*HD* hemodialysis, *SBP* systolic blood pressure, *DBP* diastolic blood pressure, *SpO*_*2*_ saturation of percutaneous oxygen, *BMI* body mass index, *PTA* percutaneous transluminal angioplasty, *FV* flow volume, *RI* resistance index, *Na* serum sodium concentration, *K* serum potassium concentration, *Ca* serum calcium concentration, *P* serum inorganic phosphate concentration, *rSO*_*2*_ regional oxygen saturation.

^#^Indicates spearman’s rank correlation for skewed distribution of data.

**Table 3:** Multiple linear regression analysis of independent factors of access-hand rSO_2_ after PTA.

Variables	Coefficient	Standardized coefficient	*P* values
Serum creatinine	1.466	0.296	0.006
Hemoglobin	3.518	0.249	0.019

## Discussion

This observational study focused on the changes in cerebral and access hand rSO_2_ before and after PTA and investigated the factors associated with access hand rSO_2_ in patients undergoing HD. Access hand rSO_2_ decreased with the increase in blood flow into the vein via the AVF by PTA, but in contrast, cerebral rSO_2_ was maintained despite significant decreases in carotid blood flow after PTA. Furthermore, serum Cr and Hb levels were associated with the maintenance of access hand oxygenation without AVF stenosis.

Previous reports confirmed the usefulness of several methods for evaluating access hand circulation in patients undergoing HD. Saturation of percutaneous oxygen (SpO_2_) measurement is one of the tissue oxygenation evaluations and its decrease in access hand was associated with the development of the steal syndrome or access-induced distal ischemia^11,12^. Skin perfusion pressure (SPP) at access hand was possible to detect ischemic steal syndrome because of the significant decreases in the amount or rate of changes in SPP after AVF creation^13^. In this study, there were significant decreases in SpO_2_ after PTA compared to that before; however, the differences were small. In addition, there was no equipment for measuring SPP in this study; therefore, we cannot directly comment on the usefulness of SPP measurement for evaluating changes in access hand circulation before and after PTA. However, access hand rSO_2_ measurement was also reported as a marker of tissue oxygenation, including the thenar muscle, and reflected the regional hemodynamic changes^10,14–16^. In addition, this instrument has the advantage of being able to measure the rSO_2_ up to four other tissues simultaneously. In this study, we aimed to investigate the influence of PTA procedure with changes in rSO_2_ values in the brain and access hand simultaneously; therefore, we performed this study using INVOS 5100c oxygen saturation monitor.

In this study, PTA for AVF with stenosis caused a decrease in access hand rSO_2_. In general, PTA improves AVF stenosis and increases the blood flow from the AVF limb artery to the vein, resulting in a decrease in circulation into the hand, including the thenal lesion, compared to before PTA. An excessive increase in blood volume into the venous system via AVF might cause ischemia in the access hand, that is, steal syndrome^4^. The symptoms could sometimes progress to severe ischemia^11^. A remarkable decrease in access hand rSO_2_ after PTA compared to that before would reflect the deterioration of access hand circulation, which is possible to lead to the occurrence of the steal syndrome. Therefore, monitoring of access hand rSO_2_ would be useful to evaluate changes in access hand circulation before and after PTA and might be possible for the early detection of the development of steal syndrome in clinical settings. In addition, when vasodilatation by PTA increases FV in the AVF limb, FV in other organs, including the brain, might decrease. Based on our experience of a case who measured cerebral and hand rSO_2_ before and after PTA^14^, PTA improved her FV at the brachial artery from 200 to 980 mL/min, and cerebral and access hand rSO_2_ decreased, which might be associated with the FV decrease to the brain by the FV increase in AVF. In addition, Kovarova et al. reported that short-time manual compression in an AVF led to an increase in cerebral oxygenation in patients undergoing HD^15^. This report showed that the presence of AVF and an increase in FV to the AVF limb might impair cerebral oxygenation. In contrast, in this study, cerebral rSO_2_ did not change after PTA. The difference between the previous^15^ and the present study was in the FV of the AVF; that is, the mean FV values were 1397 ± 822 mL/min in the previous study, in contrast to 660 ± 305 mL/min after PTA in this study. In the short-term manual compression study, brachial FV decreased by approximately 1400 mL/min during the complete interruption of blood flow by manual compression. However, the change in blood flow by PTA was about 270 mL/min (from 387 to 660 mL/min) in this study. Such differences in FV changes might affect changes in cerebral rSO_2_. In addition, changes in carotid blood flow before and after PTA were significantly smaller than those in brachial artery blood flow in this study. Polinder-Bos et al. reported that cerebral rSO_2_ did not significantly change during HD, despite decreasing cerebral blood flow^17^. Several other studies also reported that cerebral rSO_2_ did not change during HD, although patients received ultrafiltration and their blood volume was concentrated^10,18,19^. Additionally, in some reports comparing rSO_2_ between the brain and other organs under various conditions, changes in cerebral rSO_2_ were relatively small compared to oxygenation in other organs^10,20,21^. Thus, cerebral oxygenation would be maintained as the mechanism of brain autoregulation, especially if the patients’ BP remained^22^.

Tissue oxygenation evaluation was recently performed in patients undergoing HD, and rSO_2_ was investigated in the brain, liver and muscle^10,23–25^. Low tissue oxygenation may have negative influence on HD patients. Anemic conditions, overhydrated status, and hypotension could induce organ hypoxia in the brain or intra-abdominal organs^20–22,26–28^. Furthermore, cerebral oxygenation impairment could be related to cognitive impairment^29,30^, and access hand rSO_2_ was reportedly associated with grip strength^31^. Therefore, tissue hypoxia may affect the functional performance and prognosis of each organ. In this study, we analyzed the factors associated with access hand rSO_2_ after PTA, which could be an appropriate condition without stenosis as an AVF, and Hb and serum Cr levels were extracted as independently associated factors for access hand rSO_2_. Hb plays an important role as a transporter for carrying oxygen to systemic tissues, and tissue oxygenation is associated with Hb level. The maintenance of Hb level could contribute to maintaining access hand oxygenation, similar to the positive correlation between Hb levels and cerebral or hepatic rSO_2_ values^23,25^. Serum Cr is generally an indicator of kidney function and is also a metabolic product derived from the muscle. In patients with kidney dysfunction, such as HD patients, Cr would accumulate in the serum because of impaired Cr excretion from the kidney. Therefore, serum Cr levels could depend on individual muscle mass, and the serum Cr or Cr index would reportedly reflect muscle mass or sarcopenia, which involved lessened muscle mass or weakened muscle strength^32,33^. Furthermore, when patients have high muscle mass, blood volume into the muscle is high^34^.In HD patients at their forearm, an increase of muscle mass by hand exercise was associated with an increase in FV^35^, and less circulation into the muscle was related to low oxygenation^36^. Therefore, it is important to maintain muscle mass from the viewpoint of keeping peripheral circulation and tissue oxygenation, and it would be consistent that access hand rSO_2_ had a positive association with serum Cr in this study. However, we did not measure muscle mass at the AVF limb in patients included in this study; therefore, further studies are needed to confirm the association between serum Cr levels and muscle mass in patients undergoing HD.

This study had several limitations. First, the sample size was relatively small. Second, in this study, Hb and serum Cr levels were significantly associated with access hand rSO_2_ in multivariable linear regression analysis. Access hand (thenar muscle) oxygenation would be influenced by the oxygen delivery throughout regional blood flow and oxygen demand at the muscle mass. Furthermore, this study used FV at the brachial artery as a substitute for FV at the AVF limb, which may not necessarily reflect hand circulation, including that of the thenar muscle, and we could not measure the direct blood flow into the thenar muscle. Therefore, parameters with oxygen delivery and demand at access hand were missing as a confounding factor associated with access hand oxygenation in multivariable linear regression analysis in this study. Under the addition of these parameters to this analysis, the results of the significant associations between access hand rSO_2_ and each Hb and serum Cr level might change. However, numerous studies reported the thenar oxygenation using NIRS to be a valid and reliable tool in clinical settings^10,14–16,37–39^; therefore, access hand rSO_2_ evaluated in this study would reflect the actual thenar muscle oxygenation. Third, this study evaluated AVF using only the radial artery which was used for angiostomy in many HD patients. However, when the AVF is operated using a brachial artery or synthetic graft and when patients had anatomical malformations of the artery at the AVF limb, such as brachial artery bifurcation centrally, other results might be obtained regarding factors affecting access hand rSO_2_ or changes in cerebral and hand oxygenation. Finally, we could not evaluate cardiac function in the study participants. The presence of AVF would lead to non-physiological hemodynamic effects on cardiac outputs^40,41^; therefore, cardiac function or outputs might have changed before and after PTA. Thus, to clarify the factors affecting hand oxygenation and changes in cerebral oxygenation influenced by PTA procedure, further studies are needed for the future.

In conclusion, a decrease in access hand oxygenation and maintenance of cerebral oxygenation were observed throughout PTA, and access hand rSO_2_ was positively associated with Hb levels and serum Cr in patients undergoing HD. To maintain access hand oxygenation, it is important to adequately manage Hb level and maintain muscle mass, in addition to having an AVF with appropriate blood flow.

## Methods

### Patients

In this study, 85 HD patients (59 men and 26 women) who needed to perform PTA of their AVF were recruited. The causes of chronic renal failure were diabetes mellitus (50 patients), nephrosclerosis (10 patients), chronic glomerulonephritis (12 patients), and others (13 patients). Each patient received maintenance HD two or three times per week, and their HD time per session was three or four hours. AVF were anastomosed using a radial artery. We excluded patients with an AVF that was anastomosed using the ulnar artery or brachial artery and those with AVF that were anastomosed using synthetic grafts. Among the recruited patients, 60 patients had AVF on their left arm and 25 patients on their right arm. The patients’ general characteristics are summarized in Table 4. All participants signed an informed consent form to participate in this study. This study was approved by the institutional ethics committee of Hakuyukai Medical Corporation (approval number: 02–002) and performed in accordance with the provision of the Declaration of Helsinki.

**Table 4:** Characteristics of the patients.

Characteristics	Total patients (n = 85)
Male/female	59/26 (69.4/30.6)
Age (years)	74 (66–79)
HD vintage (months)	80 (31–115)
Disease	
Diabetes mellitus	50 (58.8)
Nephrosclerosis	10 (11.8)
Chronic glomerulonephritis	12 (14.1)
Others	13 (15.3)
Arteriovenous fistula	
Right arm/left arm	25 (29.4)/60 (70.6)
Medical history	
Diabetes mellitus	55 (64.7)
Hypertension	77 (90.6)
Dyslipidemia	20 (23.5)
Coronary artery disease	32 (37.6)
Cerebrovascular disease	21 (24.7)
Peripheral artery disease	17 (20.0)
Medication	
Antiplatelet agents	59 (69.4)
Anticoagulant	6 (7.1)
Antihypertensive drug	62 (72.9)
Laboratory findings	
Hemoglobin (g/dL)	11.1 ± 0.9
Albumin (g/dL)	3.5 ± 0.4
Blood urea nitrogen (mg/dL)	63.1 (51.7–71.9)
Creatinine (mg/dL)	10.2 ± 2.3
Na (mEq/L)	137.1 ± 2.7
K (mEq/L)	5.0 ± 0.7
Ca (mg/dL)	8.5 ± 0.5
P (mg/dL)	5.1 (4.6–5.7)

Categorical data are presented as number (%), continuous data are presented as mean ± standard deviation or median and interquartile range.

*HD* hemodialysis, *Na* serum sodium concentration, *K* serum potassium concentration, *Ca* serum calcium concentration, *P* serum inorganic phosphate concentration.

### Measurement

We measured blood pressure (BP), heart rate, SpO_2_ and brachial and common carotid artery blood FV with ipsilateral AVF before and after PTA. BP and heart rate were measured in the upper limb without an AVF because the AVF needed to be protected from strong pressurization. Before measurement, the patients were at rest in the supine position in the bed for at least 5 min. Blood flow was evaluated using Doppler ultrasound (Xario200; Toshiba Medical Systems Co., Tochigi, Japan) with a linear probe (12.0 MHz). One examiner measured blood flow at the vessel site with less meandering and turbulence, three times before and after PTA respectively, and used their average value as the results.

Patients’ baseline characteristics and clinical and laboratory data were collected from their medical charts. BMI was defined using dry weight. Calcium channel blockers, angiotensin converting enzyme inhibitors, angiotensin receptor blockers, β-blockers and α-blockers were defined as antihypertensive drugs.

### Cerebral and hand rSO_2_ measurements

We measured the rSO_2_ in the brain and access hand to observe the difference in each rSO_2_ before and after PTA and evaluated related factors to assess hand rSO_2_. Measured rSO_2_, which is a marker of tissue oxygenation, was monitored using by INVOS 5100c saturation monitor (Covidien Japan, Tokyo, Japan). The principles of measurement using this monitor have been previously reported^8^. Briefly, this instrument uses a light-emitting diode that transmits near-infrared light at two wavelengths (735 and 810 nm), and two silicon photodiodes that act as light detectors to measure oxygenated and deoxygenated hemoglobin (Hb) levels. The ratio of the signal strengths of oxygenated and total Hb (oxygenated + deoxygenated Hb) was calculated, and the corresponding percentage was recorded as a single numerical value that represented the rSO_2_ level^42,43^. Furthermore, the light paths leading from the emitter to the different detectors share a common part: the 30-mm detector assesses superficial tissue, whereas the 40-mm detector assesses deep tissue. By analyzing the differential signals collected by the two detectors, the rSO_2_ values in the deep tissue were obtained^44,45^. The rSO_2_ measurement sensor was attached to the patient’s forehead (cerebral rSO_2_), and the thenar muscle of the access hand (access hand rSO_2_). Thereafter, each rSO_2_ measurement was performed at rest in the supine position in the bed for at least 5 min, and mean values were defined as the measured rSO_2_ values. The attachment parts of the sensor and monitoring time of rSO_2_ were determined based on previous reports^10,12,19,27^. Measured rSO_2_ values were shown as results before and after PTA. The rSO_2_ values after PTA, which were evaluated in the state where the AVF was without stenosis, were used to evaluate the factors related to access hand rSO_2_.

### Statistics

Data are expressed as the mean ± standard deviation or median and interquartile range. To determine whether the data showed a normal distribution, we performed the Shapiro–Wilk test to evaluate each variable. The paired Student’s t-test was used for values showing normal distribution, and the Wilcoxon signed-rank test was used for values that did not show normal distribution in the comparisons of clinical parameters including various rSO_2_ and FV before and after PTA. Correlation between clinical parameters and access hand rSO_2_ after PTA were evaluated using Pearson’s correlation or Spearman’s rank correlation for data with normal and skewed distribution, respectively. Multivariate linear regression analysis was performed to identify the independent factors of access hand rSO_2_ after PTA. All analyses were performed using IBM SPSS Statistics for Windows (version 25.0; IBM Corp., Armonk, NY, USA). Statistical significance was set at p < 0.05.

### Ethics declaration

All participants provided written informed consent to participate in this study. This study was approved by the institutional ethics committee of Hakuyukai Medical Corporation (approval number: 02-002) and was performed in accordance with the provisions of the Declaration of Helsinki (as revised in Tokyo in 2004).

## Data Availability

All data analyzed during this study are available within the paper.
